# Global stability of COVID-19 model involving the quarantine strategy and media coverage effects

**DOI:** 10.3934/publichealth.2020047

**Published:** 2020-08-03

**Authors:** Ahmed A Mohsen, Hassan Fadhil AL-Husseiny, Xueyong Zhou, Khalid Hattaf

**Affiliations:** 1 Department of Mathematics, College of Education for Pure Science (Ibn Al-Haitham), University of Baghdad, Iraq; 2 Department of Mathematics, College of Science, University of Baghdad, Iraq; 3 School of Mathematics and Statistics, Xinyang Normal University, Xinyang 464000, Henan, P.R. China; 4 Centre Régional des Métiers de l'Education et de la Formation (CRMEF), 20340 Derb Ghalef, Casablanca, Morocco; 5 Laboratory of Analysis, Modeling and Simulation (LAMS), Faculty of Sciences Ben M'sik, Hassan II University of Casablanca, P.O Box 7955 Sidi Othman, Casablanca, Morocco

**Keywords:** COVID-19, media coverage effect, quarantine, mathematical modeling, backward bifurcation

## Abstract

In this paper, we build and analyze a mathematical model of COVID-19 transmission considering media coverage effects. Due to transmission characteristics of COVID-19, we can divided the population into five classes. The first class describes the susceptible individuals, the second class is exposed individuals, the third class is infected individuals, the fourth class is quarantine class and the last class is recovered individuals. The existence, uniqueness and boundedness of the solutions of the model are discussed. The basic reproduction number ${ℛ}_{0}$ is obtained. All possible equilibrium points of the model are investigated and their local stability is discussed under some conditions. The disease-free equilibrium is local asymptotically stable when ${ℛ}_{0}<1$ and unstable when ${ℛ}_{0}>1$. The globally asymptotical stability of all point is verified by Lyapunov function. Finally, numerical simulations are carried out to confirm the analytical results and understand the effect of varying the parameters on spread of COVID-19. These findings suggested that media coverage can be considered as an effective way to mitigate the COVID-19 spreading.

## 1. Introduction

In December 2019, the world is facing the emergence of a new pandemic, which is called coronavirus disease 2019 (COVID-19). Then, COVID-19 spreads to world widely over the first two months in 2020. There were 492,510 confirmed cases of COVID-19 infection and 22,185 dead cases in world [1], [2]. Therefore, it poses a continuing threat to human health because of its high transmission efficiency and serious infection consequences as well, it transmits by direct contact. Many researchers have tried to study and understand the dynamical behavior of COVID-19 through the transmission dynamics and calculate the basic reproduction number of COVID-19. It has become a key quantity to determine the spread of epidemics and control it. For example, in [3], Li et al. conducted a study of the first 425 confirmed cases in Wuhan, China, showing that the reproduction number of COVID-19 was 2.2, and revealed that person to person transmission occurred between close contacts. Other research [4] shows that the reproduction number of COVID-19 becomes 2.90, which is being increasing. In [5], Riou et al. studied pattern of early human to human transmission of COVID-19 in Wuhan, China. In [6], Hellewell et al. investigated the feasibility of controlling 2019-nCoV outbreaks by isolation of cases and contacts. Chen et al.[7], suggested mathematical model for simulation the phase-based transmissibility of novel coronavirus. Bentout et al. [8] developed an susceptible exposed infectious recovered model to estimation and prediction for COVID-19 in Algeria. Belgaid et al.[9] suggested and analysis of a model for Coronavirus spread. Owolabi et al. [10] proposed and analyzed a nonlinear epidemiological model for SARS CoV-2 virus with quarantine class. Flaxman et al. [11] suggested and estimating the effects of non-pharmaceutical interventions on COVID-19 in Europe. Kennedy et al. [12] suggested a mathematical model involving the effects of intervention strategies on COVID-19 transmission dynamics. Feng et al. [13] studied a COVID-19 model with the effects of media and quarantine in UK. In this present study, we will show effects of the quarantine strategy and media reports on the spread of COVID-19.

We propose a mathematical model for COVID-19 transmission dynamics with the quarantine strategy and media effects. We start the model formulation by denoting the total size of the population by *N* which is classified further into five classes, the susceptible *S*(*t*), the exposed *E*(*t*), the infected *I*(*t*), the hospital quarantined *Q*(*t*) and the recovery *R*(*t*) at any time *t*, So, N=S+E+I+Q+R. The exposed class means low-level virus carrier, which is considered to be non infectious. The quarantined class in which the individual who is in the process in hospital, we suppose that only those who treat it will be in contact with the infected population. Accordingly, the flow of corona virus pandemic along with the above assumptions can be representing in the following block diagram:

**Figure 1.**
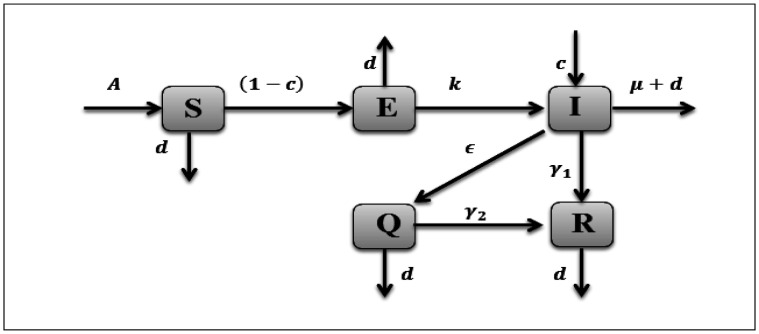
Flow diagram of the compartmental model of COVID-19.

And the corresponding dynamical model has formulated through the nonlinear differential equations as follows,

$$\begin{array}{lllll} \frac{dS(t)}{dt}=A-\left(\beta_{1}-\beta_{2}\frac{I}{m+I}\right)SI-dS, & & & & \\ \frac{dE(t)}{dt}=\left(1-c\right)\left(\beta_{1}-\beta_{2}\frac{I}{m+I}\right)SI-k\left(\beta_{1}-\beta_{2}\frac{I}{m+I}\right)EI-dE, & & & & \\ \frac{dI(t)}{dt}=c\left(\beta_{1}-\beta_{2}\frac{I}{m+I}\right)SI+k\left(\beta_{1}-\beta_{2}\frac{I}{m+I}\right)EI-\left(\varepsilon +\gamma_{1}+d+µ\right)I, & & & & \\ \frac{dQ(t)}{dt}=\varepsilon I-\left(d+\gamma_{2}\right)Q, & & & & \\ \frac{dR(t)}{dt}=\gamma_{1}I+\gamma_{2}Q-dR, & & & & \end{array}$$ (1)

with initial conditions

S(0)>0, E(0)>0, I(0)>0, Q(0)>0, R(0)>0. (2)

In model (1), the birth rate *A* is taken into susceptible class and natural death rate of population is given by the parameter *d*. The susceptible will be infected through sufficient direct contacts with infected people in the absence of media alerts by $\beta_{1}$, with fraction parameter *c*, where c∈[0,1]. The term $\beta_{2}\frac{I}{m+I}$ reduce the transmission as media continuously alert the susceptible and exposed regarding infected cases and possible preventive measures. Usually, we assume that $\beta_{1}\geq \beta_{2}$. As well, we consider the media awareness cannot stop the outbreak of COVID-19 but can aware the population to minimize the transmission risk through half saturation of media constant *m*. The death due to disease rate µ affecting from infected class only. *k* represent to a fraction denoting the level of exogenous re-infection. The quarantined rate is given by ε. And the mean recovery rates of class *I*, *Q* are $\gamma_{i},\;i=1,2$, respectively.

It is easy see that the 4*^th^* and 5*^th^* equations are a linear differential equation with respect to variables *I*(*t*) and *R*(*t*), which are not appear in the other equations of model (1). Hence model (1) can be reduced to the following model:

$$\begin{array}{lll} \frac{dS(t)}{dt}=A-\left(\beta_{1}-\beta_{2}\frac{I}{m+I}\right)SI-dS, & & \\ \frac{dE(t)}{dt}=\left(1-c\right)\left(\beta_{1}-\beta_{2}\frac{I}{m+I}\right)SI-k\left(\beta_{1}-\beta_{2}\frac{I}{m+I}\right)EI-dE, & & \\ \frac{dI(t)}{dt}=c\left(\beta_{1}-\beta_{2}\frac{I}{m+I}\right)SI+k\left(\beta_{1}-\beta_{2}\frac{I}{m+I}\right)EI-\left(\varepsilon +\gamma_{1}+d+µ\right)I. & & \end{array}$$ (3)

In this paper, we will discuss the dynamics of model (3) with initial conditions

S(0)>0,  E(0)>0,  I(0)>0. (4)

This paper is organized as follows. In section 2, we will build the basic properties of model such as (positivity, boundedness of solutions and basic reproduction number). Existence of equilibrium points is presented in section 3. In section, the phenomenon of backward bifurcation is considered. The local and global stability of equilibrium points are studied in sections 4. In section 5, numerical simulation results are given. We conclude this paper with a brief conclusion.

## 2. Basic properties of model (3)

### 2.1. Positivity of solutions

On the positivity of solutions for model (3), we have the following result.

**Theorem 2.1** Every solution of (3) with initial values (4) is positive as *t* > 0.

*Proof*. Let $t_{1}=sup\{t>0:S(t)>0,\;E(t)>0,\;I(t)>0\}>0$. It follows (3) that

$$\frac{dS(t)}{dt}=A-\left(\beta_{1}-\beta_{2}\frac{I}{m+I}\right)SI-dS,$$ (5)

which can be written as

$$\begin{array}{lll} \frac{d}{dt}\{S(t)exp[dt+\int_{0}^{t}(\beta_{1}-\beta_{2}\frac{I(\tau )}{m+I(\tau )})S(\tau )I(\tau )d\tau ]\} & & \\ \;=Aexp[dt+\int_{0}^{t}(\beta_{1}-\beta_{2}\frac{I(\tau )}{m+I(\tau )})S(\tau )I(\tau )d\tau ]. & & \end{array}$$ (6)

thus,

$$\begin{array}{lll} S(t_{1})exp[dt_{1}+\int_{0}^{t}(\beta_{1}-\beta_{2}\frac{I(\tau )}{m+I(\tau )})S(\tau )I(\tau )d\tau ]-S(0) & & \\ \;=\int_{0}^{t_{1}}Aexp[dy+\int_{0}^{y}(\beta_{1}-\beta_{2}\frac{I(\tau )}{m+I(\tau )})S(\tau )I(\tau )d\tau ]dy, & & \end{array}$$ (7)

so that

$$\begin{array}{lll} S(t_{1})=S(0)exp[-dt_{1}-\int_{0}^{{}_{1}}(\beta_{1}-\beta_{2}\frac{I(\tau )}{m+I(\tau )})S(\tau )I(\tau )d\tau ] & & \\ \;+exp[-dt_{1}-\int_{0}^{{}_{1}}(\beta_{1}-\beta_{2}\frac{I(\tau )}{m+I(\tau )})S(\tau )I(\tau )d\tau ] & & \\ \;\times \int_{0}^{t_{1}}Aexp[dy+\int_{0}^{y}(\beta_{1}-\beta_{2}\frac{I(\tau )}{m+I(\tau )})S(\tau )I(\tau )d\tau ]dy>0. & & \end{array}$$ (8)

Similarly, it can be shown that *E*(*t*) > 0 and *I*(*t*) > 0 for all time *t* > 0. Hence all solutions of the model (3) remain positive for all non-negative initial conditions, as required.

### 2.2. Boundedness

**Theorem 2.2** All solutions of model (1) which initiate in ${\mathbb{R}}_{+}^{5}$ are uniformly bounded.

*Proof*. Define the function N(t)=S(t)+E(t)+I(t)+Q(t)+R(t) and then take the time derivative of *N*(*t*) along the solution of model (1) gives $\frac{dN}{dt}\;\leq A-LN.$ Then, $\frac{dN}{dt}+LN\;\leq A,$ where L=min{d, d+µ} .

Now, it is easy to verify that the solution of the above linear differential inequalities can be written as

$$N\left(t\right)\leq \;\frac{A}{L}+(N_{0}-\frac{A}{L}\;)e^{-Lt},$$ (9)

where $N_{0}=(S\left(0\right),E\left(0\right),I\left(0\right),Q\left(0\right),R\left(0\right))$. Hence,

$$\underset{t\to \infty }{limsup}N(t)\leq \frac{A}{L}.$$ (10)

and $N(t)\leq \frac{A}{L}$ for ∀t>0. Thus all solutions are uniformly bounded and the proof is complete.

### 2.3. Basic reproduction number

It is easy to see that model (3) always has a disease-free equilibrium $P_{0}(S_{0},0,0)$, where $S_{0}=\frac{A}{d}$. We can calculate the reproduction number ${ℛ}_{0}$ of model (3) by using the next-generation matrix method illustrated by van den Driessche and Watmough in [14].

$${ℛ}_{0}=\;\frac{c\beta_{1}A}{d\left(\varepsilon +\gamma_{1}+d+µ\right)}.$$ (11)

Consequently, from Theorem 2 of [14], we have the following result.

**Theorem 2.3** The disease-free equilibrium $P_{0}$ of the model (3) is locally asymptotically when ${ℛ}_{0}<1$ and $P_{0}$ is unstable when ${ℛ}_{0}>1$.

The basic reproduction number for COVID-19 infection ${ℛ}_{0}$ measures the average number of new COVID-19 infections generated by a single infected individual in a completely susceptible population [14], [15]. Theorem 2.3 implies that COVID-19 can be eliminated from the community (when ${ℛ}_{0}<1$) if the initial sizes of the sub-populations of the model (3) are in the attraction basin of the disease-free equilibrium $P_{0}$. To ensure that COVID-19 elimination is independent of the initial sizes of the sub-populations, it is necessary to show that the disease-free equilibrium $P_{0}$ is globally asymptotically stable when ${ℛ}_{0}<1$.

## 3. Existence the COVID-19 equilibria point and backward bifurcation

In this section, we consider the number of equilibrium solutions the model (3). To do so, let $P^{*}(S^{*},E^{*},I^{*})$ be any arbitrary equilibrium of the model (3). Setting the right sides of the model (3) to zero gives

$$\begin{array}{ll} S^{*}=\frac{A}{X^{*}I^{*}+d}, & \\ E^{*}=\frac{\left(1-c\right)AX^{*}I^{*}}{\left(kXI^{*}+d)(X^{*}I^{*}+d\right)}. & \end{array}$$ (12)

here

$$X^{*}=\;\beta_{1}-\beta_{2}\frac{I^{*}}{m+I^{*}}.$$ (13)

Since we assume $\beta_{1}>\beta_{2}$, S* and E* are positive. now, substituting (12) in 3*^rd^* equation of the model (3) and simplifying it, we get

$$D_{1}I^{*4}+D_{2}I^{*3}+D_{3}I^{*2}+D_{4}I^{*}+D_{5}=0,$$ (14)

where

$$\begin{array}{lllllll} D_{1}=-k(\varepsilon +\gamma_{1}+d+µ)(\beta_{1}-\beta_{2}{)}^{2}, & & & & & & \\ D_{2}=kA{\left(\beta_{1}-\beta_{2}\right)}^{2}-(\varepsilon +\gamma_{1}+d+µ)(\beta_{1}-\beta_{2})[d(k+1)+2km\beta_{1}], & & & & & & \\ D_{3}=A(\beta_{1}+\beta_{2})(2km\beta_{1}+cd)-(\varepsilon +\gamma_{1}+d+µ)[md(k+1)(2\beta_{1}+\beta_{2})+d^{2}+k\beta_{1}^{2}m^{2}], & & & & & & \\ D_{4}=kA\beta_{1}^{2}m^{2}+cdA[\beta_{1}(m+1)-\beta_{2}-md(\varepsilon +\gamma_{1}+d+µ)(2d+m\beta_{1}), & & & & & & \\ D_{5}=d^{2}m^{2}(\varepsilon +\gamma_{1}+d+µ)({ℛ}_{0}-1). & & & & & & \end{array}$$ (15)

From (15), we can find that $D_{1}<0$. And $D_{5}>0$ when ${ℛ}_{0}>1$, $D_{5}<0$ when ${ℛ}_{0}<1$. Thus, the number of possible positive real roots the polynomial (12) can have depends on the signs of *D*_2_, *D*_3_ and *D*_4_. Let $f(x)=D_{1}x^{4}+D_{2}x^{3}+D_{3}x^{2}+D_{4}x+D_{5}$. The various possibilities for the roots of *f*(*x*) can be analyzed using the Descartes Rule of Signs. The various possibilities for the roots of *f*(*x*) are tabulated in Table 1.

**Table 1.** Number of possible positive real roots of equation (14).

Cases	*D*_1_	*D*_2_	*D*_3_	*D*_4_	*D*_5_	R_0_	Number of sign changes	Number of possible positive real roots
1	−	+	+	+	+	ℛ_0_ > 1	1	1
	−	+	+	+	−	ℛ_0_ < 1	2	0,2
2	−	+	+	−	+	ℛ_0_ > 1	3	1,3
	−	+	+	−	−	ℛ_0_ < 1	2	0,2
3	−	+	−	+	+	ℛ_0_ > 1	3	1,3
	−	+	−	+	−	ℛ_0_ < 1	4	0,2,4
4	−	+	−	−	+	ℛ_0_ > 1	3	1,3
	−	+	−	−	−	ℛ_0_ < 1	2	0,2
5	−	−	+	+	+	ℛ_0_ > 1	1	1
	−	−	+	+	−	ℛ_0_ < 1	2	0,2
6	−	−	+	−	+	ℛ_0_ > 1	3	1,3
	−	−	+	−	−	ℛ_0_ < 1	2	0,2
7	−	−	−	+	+	ℛ_0_ > 1	1	1
	−	−	−	+	−	ℛ_0_ < 1	2	0,2
8	−	−	−	−	+	ℛ_0_ > 1	1	1
	−	−	−	−	−	ℛ_0_ < 1	0	0

**Theorem 3.1** The model (3)

(i) has a unique endemic equilibrium if ${ℛ}_{0}>1$ and whenever Cases 1, 5, 7 and 8 are satisfied;

(ii) could have more than one endemic equilibrium if ${ℛ}_{0}>1$ and Cases 2, 3, 4 and 6 are satisfied;

(iii) could have 2 or more endemic equilibria if ${ℛ}_{0}<1$ and Cases 1–7 are satisfied.

From the 4*^th^* and 5*^th^* equations of model (1) we can determent the values of Q* and R* through

$$\begin{array}{c} Q^{*}=\frac{\varepsilon I^{*}}{d+\gamma_{2}},\\ R^{*}=\frac{\gamma_{1}I^{*}+\gamma_{2}Q^{*}}{d}. \end{array}$$ (16)

The existence of multiple endemic equilibria when ${ℛ}_{0}<1$ suggests the possibility of backward bifurcation [16], where the stable disease-free equilibrium co-exists with a stable endemic equilibrium when ${ℛ}_{0}<1$. This is can be obtained using Centre Manifold Theory.

**Theorem 3.2** The model (3) exhibits backward bifurcation whenever $m>\frac{{\left(1-c\right)}^{2}A\beta_{2}}{d^{2}}$ and no backward bifurcation otherwise.

*Proof*. To prove existence of backward bifurcation in the model (3) the Center Manifold approach as outlined by Castillo-Chavez and Song in [17] is used.

Firstly, for clarity and understanding of the Center Manifold Theory the model (3) variables are transformed as follows $x_{1}=S,\;x_{2}=E,\;x_{3}=I$. Define $X=(x_{1},x_{2},x_{3}{)}^{Τ}$ (Τ denotes transpose), such that the model (3) can be rewritten as $\frac{dX}{dt}=F(X)$ where $F=(f_{1},f_{2},f_{3}).$ Hence,

$$\begin{array}{lll} \frac{dx_{1}(t)}{dt}=f_{1}=A-\left(\beta_{1}-\beta_{2}\frac{x_{3}}{m+x_{3}}\right)x_{1}x_{3}-dx_{1}, & & \\ \frac{dx_{2}(t)}{dt}=f_{2}=\left(1-c\right)\left(\beta_{1}-\beta_{2}\frac{x_{3}}{m+x_{3}}\right)x_{1}x_{3}-k\left(\beta_{1}-\beta_{2}\frac{x_{3}}{m+x_{3}}\right)x_{2}x_{3}-dx_{2}, & & \\ \frac{dx_{3}(t)}{dt}=f_{3}=c\left(\beta_{1}-\beta_{2}\frac{x_{3}}{m+x_{3}}\right)x_{1}x_{3}+k\left(\beta_{1}-\beta_{2}\frac{x_{3}}{m+x_{3}}\right)x_{2}x_{3}-\left(\varepsilon +\gamma_{1}+d+µ\right)x_{3}. & & \end{array}$$ (17)

Now let $\beta_{1}=\beta_{1}^{*}$ be the bifurcation parameter. Observe that at ${ℛ}_{0}=1$,

$$\beta_{1}=\beta_{1}^{*}=\frac{d\left(\varepsilon +\gamma_{1}+d+µ\right)}{cA}.$$ (18)

With $\beta_{1}=\beta_{1}^{*}$ the transformed model equation (17) has a simple eigenvalue with zero real part and all other eigenvalues are negative (that is has a hyperbolic equilibrium point). Thus, Center Manifold Theory can be applied to investigate the local dynamics of the transformed system (17) near $\beta_{1}=\beta_{1}^{*}$. Now the Jacobian matrix of the transformed system evaluated at COVID-19 free equilibrium $P_{0}$ is obtained as

$$J(P_{0})=\;\left(\begin{array}{ccc} -d & 0 & \beta_{1}S_{0}\\ 0 & -d & (1-c)\beta_{1}S_{0}\\ 0 & 0 & c\beta_{1}S_{0}-(\varepsilon +\gamma_{1}+d+µ) \end{array}\right).$$ (19)

It is easy to obtain the right eigenvectors of this Jacobian matrix as $V=(v_{1},v_{2},v_{3}{)}^{Τ}$, where ${\left(v_{1},v_{2},v_{3}\right)}^{Τ}=(\frac{\beta_{1}S_{0}}{d},\frac{(1-c)\beta_{1}S_{0}}{d},1)$. Similarly, it is possible to obtain the left eigenvectors which are denoted by $W=(w_{1},w_{2},w_{3})=(0,0,1)$. Now proceeding to obtain the bifurcation coefficients **a** and **b** as defined in Theorem 4.1 in [17].

First the non-vanishing partial derivatives of the transformed model (17) evaluated at COVID-19 free equilibrium are obtained as

$$\frac{\partial^{2}f_{1}}{\partial x_{1}\partial x_{3}}=\frac{\partial^{2}f_{1}}{\partial x_{3}\partial x_{1}}=\frac{m}{\beta_{2}}-\beta_{1},\;\frac{\partial^{2}f_{1}}{\partial x_{3}^{2}}=\frac{2\beta S_{0}}{m},$$ (20)

$$\begin{array}{cc} \frac{\partial^{2}f_{2}}{\partial x_{1}\partial x_{3}}=\frac{\partial^{2}f_{2}}{\partial x_{3}\partial x_{1}}=(1-c)\beta_{1},\;\frac{\partial^{2}f_{2}}{\partial x_{2}\partial x_{3}}=\frac{\partial^{2}f_{2}}{\partial x_{3}\partial x_{2}}=-k\beta_{1}, & \\ \;\frac{\partial^{2}f_{2}}{\partial x_{3}^{2}}=-2(1-c)\frac{\beta_{2}S_{0}}{m},\frac{\partial^{2}f_{3}}{\partial x_{1}\partial x_{3}}=\frac{\partial^{2}f_{3}}{\partial x_{3}\partial x_{1}}=c\beta_{1},\;\frac{\partial^{2}f_{3}}{\partial x_{3}^{2}}=-\frac{2c\beta_{2}}{m}, & \end{array}$$ (21)

so that

$$\begin{array}{lll} a=\sum_{k,i,j=1}^{3}w_{k}v_{i}v_{j}\frac{\partial^{2}f_{k}}{\partial x_{i}\partial x_{j}} & & \\ \;=2w_{3}v_{1}v_{3}\frac{\partial^{2}f_{3}}{\partial x_{1}\partial x_{3}}+w_{3}v_{3}^{2}\frac{\partial^{2}f_{3}}{\partial x_{3}^{2}} & & \\ \;=\frac{2c\beta_{1}^{2}S_{0}}{d^{2}}(1-\frac{{\left(1-c\right)}^{2}\beta_{2}S_{0}}{dm}). & & \end{array}$$ (22)

The sign of the bifurcation parameter **b** is associated with the following non-vanishing partial derivatives of *F*(*X*), also evaluated at the disease free equilibrium $P_{0}$:

$$\frac{\partial^{2}f_{1}}{\partial x_{3}\partial \beta_{1}}=-S_{0},\;\frac{\partial^{2}f_{2}}{\partial x_{3}\partial \beta_{1}}=(1-c)S_{0},\;\frac{\partial^{2}f_{3}}{\partial x_{3}\partial \beta_{1}}=cS_{0}.$$ (23)

The bifurcation coefficient **b** is obtained as

$$\begin{array}{lll} b=\sum_{k,i=1}^{3}v_{k}w_{i}\frac{\partial^{2}f_{k}}{\partial x_{i}\partial \beta_{1}} & & \\ \;=v_{1}w_{3}\frac{\partial^{2}f_{1}}{\partial x_{3}\partial \beta_{1}}+v_{2}w_{3}\frac{\partial^{2}f_{2}}{\partial x_{3}\partial \beta_{1}}+v_{3}w_{3}\frac{\partial^{2}f_{3}}{\partial x_{3}\partial \beta_{1}} & & \\ \;=cS_{0}(1+\frac{(2-c\beta_{1})A}{d^{2}})>0. & & \end{array}$$ (24)

Obviously, **b** is always positive. From Theorem 3.2 the system (17) will exhibit backward bifurcation phenomena if the bifurcation coefficient **a** is positive. The positivity of **a** in (22) gives the condition for backward bifurcation, which leads to

$$m>\frac{{\left(1-c\right)}^{2}A\beta_{2}}{d^{2}}.$$ (25)

## 4. Stability analysis

In this section, the stability analysis of the all equilibrium points of model (3) studied as shown in the following theorems by used some criterion.

**Theorem 4.1** The COVID-19 equilibrium point P* of the model (3) is locally asymptotically if the following conditions are hold

$$\beta_{2}\frac{I^{*}\left(2m+I^{*}\right)}{{\left(m+I^{*}\right)}^{2}}\;<\;\beta_{1},$$ (26)

$$\left[\left(\beta_{1}-\beta_{2}\frac{I^{*}(2m+I^{*})}{{\left(m+I^{*}\right)}^{2}}\right)\left(\left(1-c\right)S^{*}-kE^{*}\right)\right]+\frac{d(d-1+k)}{k}<\;XI^{*}<\frac{cd}{k\left(1-c\right)-c}.$$ (27)

*Proof*. The Jacobian matrix of model (3) at $P^{*}\;$ can be written as

$$J(P^{*})=\left(\begin{array}{ccc} a_{11} & 0 & a_{13}\\ a_{21} & a_{22} & a_{23}\\ a_{31} & a_{32} & 0 \end{array}\right),$$ (28)

here

$$\begin{array}{cccc} a_{11}=-(XI^{*}+d\;),a_{13}=\frac{\beta_{2}S^{*}I^{*}\left(2m+I^{*}\right)}{{\left(m+I^{*}\right)}^{2}}-\beta_{1}S^{*},\; & & & \\ a_{21}=\left(1-c\right)XI^{*},a_{22}=\;-(kXI^{*}+d),\; & & & \\ a_{23}=\left(1-c\right)\left(\beta_{1}S^{*}-\frac{\beta_{2}S^{*}I^{*}\left(2m+I^{*}\right)}{{\left(m+I^{*}\right)}^{2}}\right)-k\left(\beta_{1}E^{*}-\frac{\beta_{2}E^{*}I^{*}\left(2m+I^{*}\right)}{{\left(m+I^{*}\right)}^{2}}\right), & & & \\ a_{31}=cXI^{*},a_{32}=kXI^{*}. & & & \end{array}$$ (29)

clearly, the characteristics equation of *J*(P*) is given by

$$\lambda^{3}+B_{1}\lambda^{2}+B_{2}\lambda +B_{3}=0,$$ (30)

where

$$\begin{array}{ccc} B_{1}=-\left[a_{11}+a_{22}\right],\; & & \\ B_{2}=a_{11}a_{22}-a_{13}a_{31}-a_{23}a_{32},\; & & \\ B_{3}=-\left[a_{11}\left(-a_{23}a_{32}\right)+a_{13}(a_{21}a_{32}-a_{22}a_{31})\right]. & & \end{array}$$ (31)

furthermore, we have that

$$\begin{array}{cc} \Delta =\;B_{1}B_{2}-B_{3}\; & \\ =-a_{11}a_{22}\left[a_{11}+a_{22}\right]+a_{11}a_{13}a_{31}+a_{22}a_{23}a_{32}+a_{13}a_{21}a_{32}. & \end{array}$$ (32)

Now, according to Routh-huewitz criterion P* will be locally asymptotically stable provided that $B_{1}>0,\;B_{3}>0$ and Δ>0. It is clear that if above conditions (26)–(27) hold.

The purpose of this section is to investigate the global stability by using Lyapunov function for COVID-19 free equilibrium point and COVID-19 equilibrium point respectively. We obtain the result in the following theorems

**Theorem 4.2** The disease-free equilibrium $P_{0}$ is globally asymptotically stable provided that the following condition holds:

$$\frac{{ℛ}_{0}}{c}\;<1.$$ (33)

*Proof*. Consider the following function

$$V_{0}\left(S,E,I\right)=\;\left(S-S_{0}-S_{0}\text{ln}\frac{S}{S_{0}}\;\right)+E+I.$$ (34)

clearly, $V_{0}:\;{\mathbb{R}}_{+}^{3}\to \mathbb{R}$ is a continuously differentiable function such that $V_{0}\left(S_{0},\;0,\;0\right)=0\;$ and $V_{0}\left(S,\;E,I\right)>0,\;\forall \;\left(S,\;E,I\right)\neq \left(S_{0},\;0,\;0\right)$. Further, we have

$$\begin{array}{ccc} \frac{dV_{0}}{dt}=\;\left(\frac{S-S_{0}}{S}\right)\left[A-\left(\beta_{1}-\beta_{2}\frac{I}{m+I}\right)SI-dS\right]\; & & \\ +\left[\left(1-c\right)\left(\beta_{1}-\beta_{2}\frac{I}{m+I}\right)SI-k\left(\beta_{1}-\beta_{2}\frac{I}{m+I}\right)EI-dE\right]\; & & \\ +\left[c\left(\beta_{1}-\beta_{2}\frac{I}{m+I}\right)SI+k\left(\beta_{1}-\beta_{2}\frac{I}{m+I}\right)EI-(\varepsilon +\gamma_{1}+d+µ)I\right]. & & \end{array}$$ (35)

now, by doing some algebraic manipulation and using the condition (33), we get

$$\frac{dV_{0}}{dt}\;\leq \;-\frac{d}{S}{\left(S-S_{0}\right)}^{2}-\beta_{2}S_{0}\frac{I^{2}}{m+I}-dE-\left[\left(\varepsilon +\gamma_{1}+d+µ\right)-\beta_{1}S_{0}\right]I.$$ (36)

Obviously, $dV_{0}/dt=0$ at $P_{0}=(S,0,0)$, moreover $dV_{0}/dt<0$ otherwise. Hence $dV_{0}/dt$ is negative definite and then the solution starting from any initial point satisfy the condition (33), will approaches asymptotically to COVID-19 free equilibrium point. Hence the proof is complete.

**Theorem 4.3** P* in case *i* of Th. (3.1) is globally asymptotically stable if ${ℛ}_{0}>1$.

*Proof*. At the COVID-19 equilibrium point $P^{*}=(S^{*},\;E^{*},\;I^{*}),S^{*},E^{*}$ and I* satisfies the following equations

$$\begin{array}{lll} A-\left(\beta_{1}-\beta_{2}\frac{I}{m+I}\right)SI-dS=0, & & \\ (1-c)\left(\beta_{1}-\beta_{2}\frac{I}{m+I}\right)SI-k\left(\beta_{1}-\beta_{2}\frac{I}{m+I}\right)EI-dE=0, & & \\ c\left(\beta_{1}-\beta_{2}\frac{I}{m+I}\right)SI+k\left(\beta_{1}-\beta_{2}\frac{I}{m+I}\right)EI-(\varepsilon +\gamma_{1}+d+µ)I=0 & & \end{array}$$ (37)

By above equations (4.4) and assumptions

$$\frac{S}{S^{*}}=x,\;\frac{E}{E^{*}}=y,\;\frac{I}{I^{*}}=u$$

we obtian

$$\begin{array}{lll} \dot{x}=x\left[\frac{A}{S^{*}}\left(\frac{1}{x}-1\right)-\beta_{1}I^{*}(u-1)+\frac{\beta_{2}I^{2}{}^{*}}{m+I^{*}}\left(\frac{u^{2}(m+I^{*})}{m+I}-1\right)\right] & & \\ \dot{y}=y\left\{(1-c)\left[\frac{\beta_{1}S^{*}I^{*}}{E^{*}}\left(\frac{xu}{y}-1\right)-\frac{\beta_{2}S^{*}I^{2}{}^{*}}{(m+I^{*})E^{*}}\left(\frac{(m+I^{*})xu^{2}}{(m+I)y}-1\right)\right]-k\beta_{1}I^{*}(u-1)+\frac{k\beta_{2}I^{2}{}^{*}}{m+I^{*}}\left(\frac{(m+I^{*})u^{2}}{m+I}-1\right)\right\} & & \\ \dot{u}=u\left[c\beta_{1}S^{*}(x-1)-\frac{c\beta_{2}S^{*}I^{*}}{m+I^{*}}\left(\frac{(m+I^{*})xu}{m+I}-1\right)+k\beta_{1}E^{*}(y-1)-\frac{k\beta_{2}E^{*}I^{*}}{m+I^{*}}\left(\frac{(m+I^{*})yu}{m+I}-1\right)\right] & & \end{array}$$ (38)

now, define the Lyapunov function

$$V_{1}=S^{*}(x-1-lnx)+E^{*}(y-1-lny)+I^{*}(u-1-lnu)$$ (39)

clearly, by derivative of $V_{1}$ we get

$$\frac{dV_{1}}{dt}=S^{*}\frac{x-1}{x}\dot{x}+E^{*}\frac{y-1}{y}\dot{y}+I^{*}\frac{u-1}{u}\dot{u}$$

$$\begin{array}{llll} \frac{dV_{1}}{dt}=(x-1)\left[A\left(\frac{1}{x}-1\right)-\beta_{1}S^{*}I^{*}(u-1)+\frac{\beta_{2}S^{*}I^{2}{}^{*}}{m+I^{*}}\left(\frac{u^{2}(m+I^{*})}{m+I}-1\right)\right] & & & \\ \;+(y-1)\left\{(1-c)\left[\beta_{1}S^{*}I^{*}\left(\frac{xu}{y}-1\right)-\frac{\beta_{2}S^{*}I^{2}{}^{*}}{m+I^{*}}\left(\frac{(m+I^{*})xu^{2}}{(m+I)y}-1\right)\right]+\frac{k\beta_{2}E^{*}I^{2}{}^{*}}{m+I^{*}}\left(\frac{(m+I^{*})u^{2}}{m+I}-1\right)\right\} & & & \\ \;+(u-1)\left[c\beta_{1}S^{*}I^{*}(x-1)-\frac{c\beta_{2}S^{*}I^{2}{}^{*}}{m+I^{*}}\left(\frac{(m+I^{*})xu}{m+I}-1\right)-\frac{k\beta_{2}E^{*}I^{2}{}^{*}}{m+I^{*}}\left(\frac{(m+I^{*})yu}{m+I}-1\right)\right] & & & \end{array}$$

furthermore, by simplifying the resulting terms, we get that

$$\begin{array}{lll} =A[2-x-\frac{1}{x}]+\beta_{1}S^{*}I^{*}[x+u-c(x+u)-(1-c)(y+\frac{xu}{y})] & & \\ \;-\frac{\beta_{2}S^{*}I^{2}{}^{*}}{m+I^{*}}\left[x-y(1-c)-cu+(u^{2}-(1-c)\frac{xu^{2}}{y}-cxu)\left(\frac{m+I^{*}}{m+I}\right)\right] & & \\ \;-\frac{k\beta_{2}E^{*}I^{2}{}^{*}}{m+I^{*}}\left[y-u+(u^{2}-uy)\left(\frac{m+I^{*}}{m+I}\right)\right] & & \end{array}$$

Since the arithmetical mean is greater than, or equal to the geometrical mean, then $2-x-\frac{1}{x}\leq 0$ for *x*>0 and $2-x-\frac{1}{x}=0$ if and only if *x*=1; $x+u-c(x+u)-(1-c)(y+\frac{xu}{y})\leq 0$ for *x*,*y*,*u*>0 and $x+u-c(x+u)-(1-c)(y+\frac{xu}{y})=0$ if and only if x=y=u=1; $y-u+(u^{2}-uy)\left(\frac{m+I^{*}}{m+I}\right)\leq 0$ for *y*,*u*>0 and $y-u+(u^{2}-uy)\left(\frac{m+I^{*}}{m+I}\right)=0$ if and only if *y*=*u*=1. Therefore, ${\dot{V}}_{1}\leq 0$ for *x*,*y*,*u*>0 and ${\dot{V}}_{1}=0$ if and only if x=y=u=1, the maximum invariant set of model (3) on the set $\left\{(x,y,u):{\dot{V}}_{1}=0\right\}$ is the singleton (1,1,1). Thus, the COVID-19 equilibrium point P* is globally asymptotically stable if ${ℛ}_{0}>1$,by LaSalle Invariance Principle [18]. Hence, the proof is complete.

## 5. Numerical simulation

For the parameters values of model (1.1), we can chosen the parameters values from real data available sense Feb. 24 2020 to Apr. 5 2020. The total population of the Iraq for the year 2020 is approximately 40 × 10^6^ [19]. The life expectancy in Iraq is approximatily 71.08 [19]. Clearly, we can obtain that the natural death rate $d=3.8545\times 10^{-5}$ per day. The birth rate is estimated from *A*/*d*=*N*, and assumed that this is to be the bound population in the disease absence. So, *A*=1541.8 per day and the other parameters of our model shows that in Table 2.

**Table 2.** Definitions and values of model parameters.

Parameter	Definition	Value	Source
A	Birth rate	1541.8	[19]
*β*_1_	Transmission contact rate between *S* and *I*	0.5	Estimated
c	Fraction constant	[0–1]	Estimated
*β*_2_	Awareness rate	0.1	Estimated
m	Half saturation of media constant	70	Estimated
d	Natural death rate	3.854510^−5^	[19],[20]
k	Fraction denoting the level of exogenous re-infection	0.05	Estimated
*ε*	Quarantined rate	1/7	[13]
*γ*_1_	Recovery rate from infected wihout quarantin strategy	0.033	Estimated
*γ*_2_	Recovery rate from quarantin class	1/18	[13]
*µ*	Death due to disease rate	0.38	[19]

**Figure 2.**
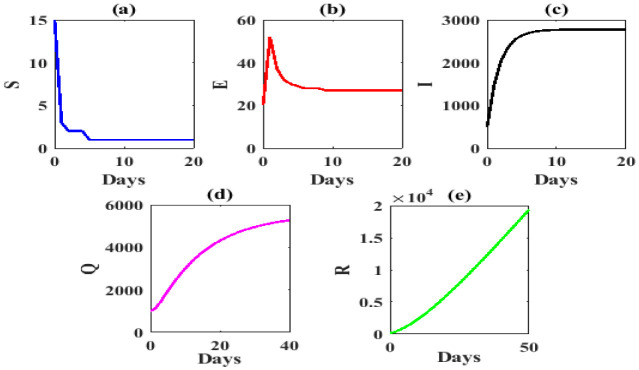
Solution trajectories converge to COVID-19 equilibrium point P*=(1,27,2773,5428,19371), by parameter value in Table 2.

We plot the solution trajectories of model (1) with initial point (15,20,500,1000,150) which converges to COVID-19 equilibrium point P*=(1,27,2773,5428,19371), shown that in Figure 2.

**Table 3.** Different government control measures and corresponding *β*_1_ values.

No.	Date	Government measures	*β*_1_
1	Feb. 24 2020	(1) detection of the first case of COVID-19 in Iraq	0.3
		(2) quarantined as preliminary control	
2	Feb. 25 2020	(1) medical examination for all individuals who are in contact with the affected case	0.1
		(2) cancellation of some mass gatherings	
		(3) increase the awareness programs about prevention measures	
3	Feb. 25-Mar. 24 2020	(1) cancellation of all religious and social events throughout Iraq	0.09
		(2) preventing movement between all provinces	
		(3) the suspension of attendance at universities and schools	
		(4) providing a number of hospitals to be places for prevention confirmed cases	
4	Mar. 24-Apr. 5 2020	(1) close all borders with neighboring countries	0.08
		(2) to declare a state of emergency and impose a curfew	
		(3) medical support from the government	
		(4) methodological improvement on the diagnosis and treatment strategy	
		(5) spontaneous household quarantine by citizens	
		(6) more newly-hospitals put into use	
		(7) massive online teaching in postponed semester	
		(8) addition of new diagnosis method clinically diagnosis in Baghdad and some provinces	

In the face of the COVID-19 outbreak, many stringent measures were taken by Iraqi government present in Table 3, to simulate the impact of different government control measures on the number of all S(t),E(t),I(t),Q(t) and *R*(*t*). We assumed that some values to contact rates with awareness Table 3, with the other parameters in Table 2 staying still on the all stages.

**Figure 3.**
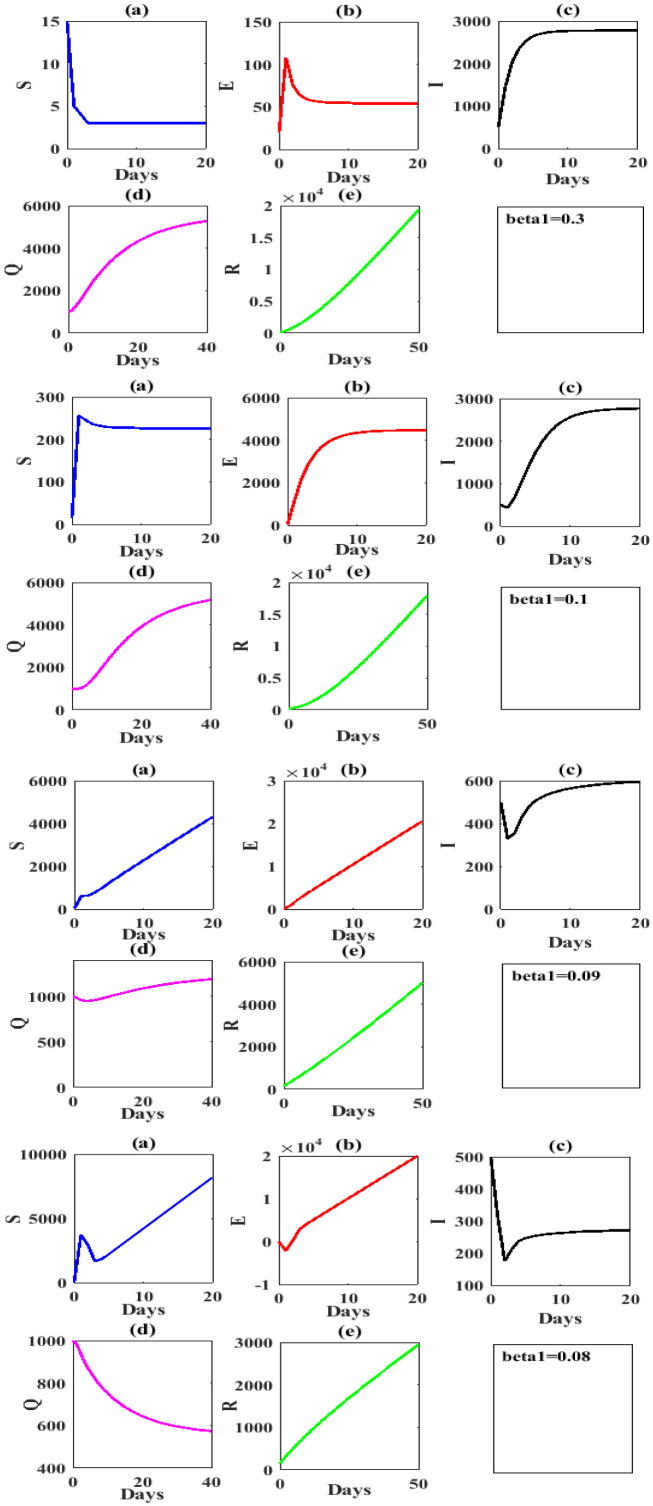
Time series to value simulation curve of different values of contact rates *β*_1_=0.3,0.1,0.09,0.08 respectively with keeping other parameters values are taken in Table 2.

The following Figure 3 shows the values of S(t),E(t),I(t),Q(t) and *R*(*t*) under government measures that above to control of COVID-19 outbreak.

**Figure 4.**
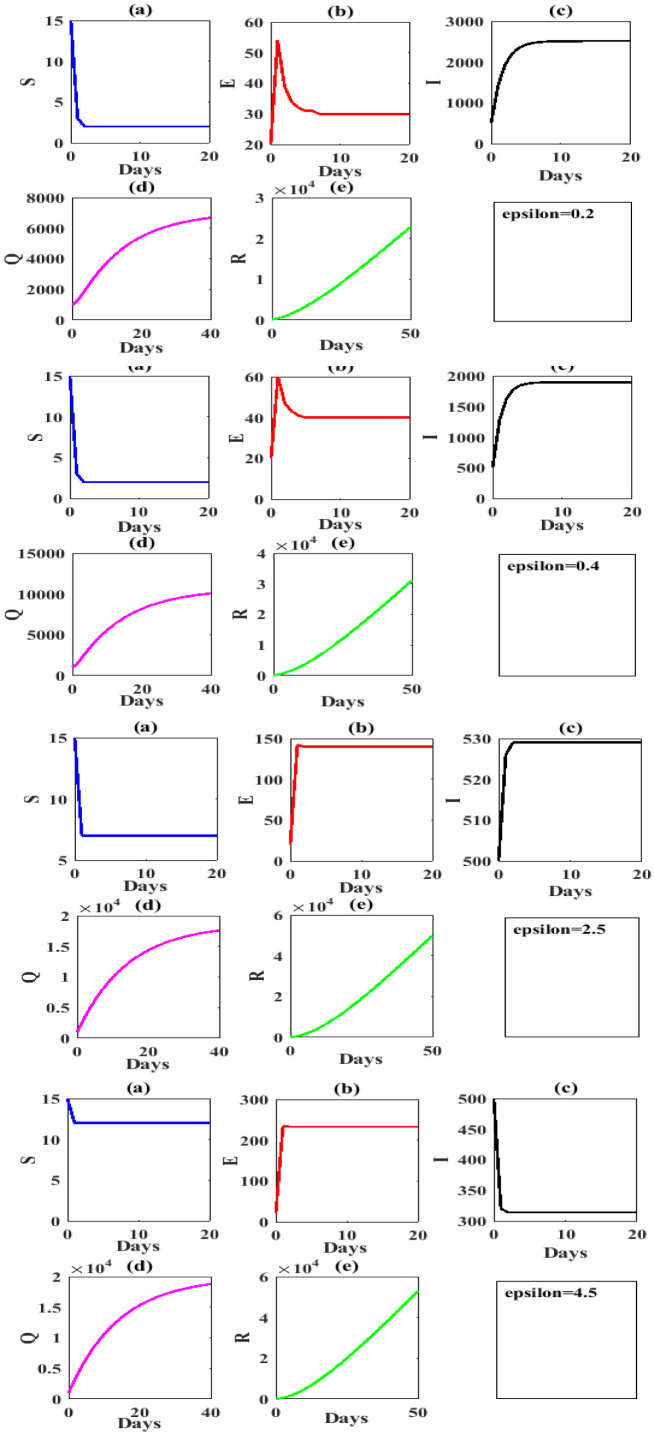
Time series to value simulation curve of different values of quarantined rates *ε*=0.2,0.4,2.5,4.5 respectively with keeping other parameters values are taken in Table 2.

Clearly, from above figure for effect of contact rate Table 3, We obtain that in case decrease the contact rate (social isolation) the reproduction number less than one and the dynamical behavior of model (1.1) still approaches to COVID-19 equilibrium point. Hence, the backward bifurcation is occur. Now, to investigate the effect of the quarantined strategy it is given by *ε* on the dynamical behavior of model (1.1) and to control to COVID-19 outbreak in Iraq. We study the impact of this parameter on values of S(t),E(t),I(t),Q(t) and *R*(*t*) in follows Table 4 and shows the results in Figure 4.

**Table 4.** Different government control measures and corresponding *ε* values.

No.	Date	Government measures	*ε*
1	Feb. 24 2020	(1) quarantined as preliminary control in Iraq	0.2
2	Feb. 25 2020	(1) medical examination for all individuals who are in contact with the affected case	0.4
		(2) cancellation of some mass gatherings	
		(3) increase the awareness programs about prevention measures	
3	Feb. 25-Mar. 24 2020	(1) direct the media to explain the symptoms of the epidemic	2.5
		(2) Preventing movement between all provinces	
		(3) Providing a number of hospitals to be places for prevention confirmed cases	
4	Mar. 24-Apr. 5 2020	(1) to declare a state of emergency and impose a curfew to reduce the contact between people	4.5
		(2) medical support from the government	
		(3) methodological improvement on the diagnosis and treatment strategy	
		(4) spontaneous household quarantine by citizens	
		(5) addition of new diagnosis method clinically diagnosis in Baghdad and some provinces	

The following Figure 4 shows the values of S(t),E(t),I(t),Q(t) and *R*(*t*) under government measures that above to control of COVID-19 outbreak.

**Figure 5.**
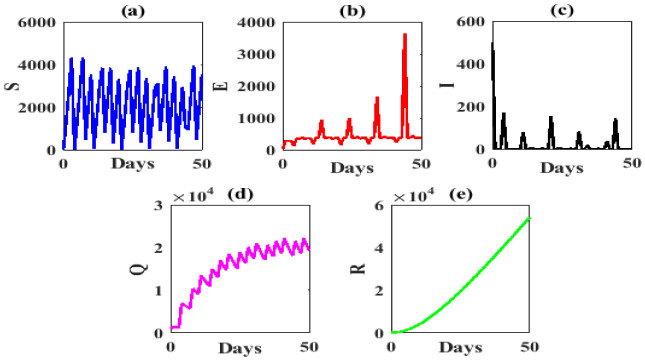
Time series to value simulation curve of different values of quarantined rates 20.5 ≤ *ε* ≤ 30.5. With keeping other parameters values are taken in Table 2.

Clearly, from above investigate to impact of the quarantined strategy Table 4, when the quarantine strategy increasing we get the number of infected is decrease and other classes are increase. Here, we ask whether the quarantine strategy is the best solution? The answer is possible, but for specific numbers. Whereas, if the quarantine is more than the capacity of the health institutions. We get the dynamical behavior of model (1.1) lose the stability as shown in Figure 5.

## 6. Discussion and results

In this research, a mathematical model of COVID-19 transmission has been proposed by compartment the total population into five epidemiological status, namely, susceptible *S*(*t*), exposed *E*(*t*), infected *I*(*t*), quarantine *Q*(*t*) and recovered *R*(*t*). The model incorporates the impact of social awareness programs conducted by public health officials with quarantine strategy in hospital. It has been noticed that these awareness programs and quarantine strategy result in human behavioral changes in order to avoid risk of disease transmission. The model mainly accounts for the reduction in disease class due to awareness. While we can say the disease goes away due to applied the quarantine it well. The proposed model has two biological equilibrium points are COVID-19 free and COVID-19. The COVID-19 free has been local stability when ${ℛ}_{0}<1$. Otherwise when ${ℛ}_{0}>1$, the COVID-19 free point becomes unstable and the dynamical behavior of the model converges to COVID-19 equilibruim point. The backward bifurcation occur if ${ℛ}_{0}=1$ at the parameter bifurcation $\beta_{1}=\beta_{1}^{*}=d\left(\varepsilon +\gamma_{1}+d+µ\right)/cA$. As well as the different government control measures have been also discussed. Furthermore, to shown and understand the effect of quarantine rate of disease we have choosed many different value of it say parameter then we have obtained some different results see Table 4 and Figure 4.
